# Supplementary material for: Quantitative PCR (qPCR) assay for the specific detection of the Chinese mystery snail (Cipangopaludina chinensis) in the UK
Source: PLoS One. 2023 Oct 5;18(10):e0292163. doi: 10.1371/journal.pone.0292163 (PMC10553251; doi:10.1371/journal.pone.0292163)
Supplement: S1 File — (DOCX) [file pone.0292163.s001.docx]

# Supporting information 1. Sample/Specimen Information

**S1 Table. Pevensey Levels Ditch Water Samples 2021.**

| **Sample ID** | **GPS Location** | **Collection date/time** | **Volume filtered** | **Turbidity of sample** | **Site conditions** |
| --- | --- | --- | --- | --- | --- |
| **EASTERN SECTION DITCH** | | | | | |
| **1-3 pool - 1** | TQ 61255 07046 | 10/08/2021 13.30 | 230ml | medium | sunny/breeze |
| **1-3 pool - 2** | TQ 61283 07016 | 10/08/2021 13.50 | 1-150ml  2-150ml | high | sunny/breeze |
| **4** | TQ 61223 07068 | 10/08/2021 13.25 | 130ml | high | sunny/breeze |
| **5** | TQ 61140 07129 | 10/08/2021 14.00 | 250ml | low | sunny/breeze |
| **6** | TQ 61109 07140 | 10/08/2021 15.00 | 260ml | medium | sunny/breeze |
| **6-7^#^** | TQ 61081 07154 | 10/08/2021 15.30 | 400ml | low | sunny/breeze |
| **7** | TQ 61035 07169 | 10/08/2021 11.00 | 1-100ml  2-100ml | high | sunny/breeze |
| **8** | TQ 60918 07188 | 10/08/2021 09.30 | 500ml | low | cloudy/breeze |
| **8-18 lower side** | TQ 60930 07153 | 10/08/2021 10.15 | 500ml | medium | cloudy/breeze |
| **8-18 upper side** | TQ 60946 07184 | 10/08/2021 10.10 | 200ml | medium, difficult access | cloudy/breeze |
| **18** | TQ 60969 07172 | 10/08/2021 10.25 | 300ml | medium | sunny/breeze |
| **18-7 lower*** | TQ 61012 07158 | 10/08/2021 10.45 | 400ml | low | cloudy/breeze |
| **18-7 upper** | TQ 61012 07173 | 10/08/2021 10.45 | 250ml | medium | sunny/breeze |
| **WESTERN SECTION DITCH** | | | | | |
| **12** | TQ 60911 07185 | 10/08/2021 09.30 | 1-118ml  2-100ml | medium | cloudy/breeze |
| **17-16** | TQ60879 07206 | 11/08/2021 09.25 | 1-170ml  2-170ml | medium, slight colouration | cloudy |
| **16** | TQ60839 07224 | 11/08/2021 09.55 | 1-150ml  2-150ml | low | cloudy/sunny |
| **End of barn at oak tree* (near 16)** | TQ60848 07217 | 12/08/2021 12.45 | 150ml | med, lots of dick weed | sunny intervals, warm |
| **15** | TQ60806 07234 | 11/08/2021 10.20 | 1- 90ml  2- 80ml | high, pink colouration | sunny intervals, warm |
| **11 opposite ditch C** | TQ60724 07250 | 11/08/2021 10.50 | 250ml | low | sunny, warm |
| **9 opposite ditch D** | TQ60669 07245 | 11/08/2021 11.25 | 300ml | low | sunny, some cloud |
| **13 opposite scape in field** | TQ60632 07216 | 11/08/2021 11.35 | 1-140ml  2-140ml | medium, ditch scrubbed over, reeds, difficult access, some colouration | sunny, some cloud |
| **SIDE DITCHES** | | | | | |
| **4-J (between 4 and ditch J)** | TQ 61221 07040 | 10/08/2021 14.50 | 350ml | low | sunny/breeze |
| **Ditch J** | TQ 61186 06962 | 10/08/2021 15.00 | 265ml | medium | sunny/breeze |
| **Ditch A** | TQ 61435 06975 | 10/08/2021 14.10 | 230ml | medium | sunny/breeze |
| **Ditch G lower (near 12)** | TQ 60907 07168 | 10/08/2021 12.10 | 1-70ml 2-80ml | high, very fine sediment | sunny/breeze |
| **Ditch G upper above culvert** | TQ 60899 07130 | 10/08/2021 12.15 | 290ml | medium | sunny/breeze |
| **Ditch H lower section (near 7)** | TQ 61031 07138 | 10/08/2021 11.40 | 250ml | high | sunny/breeze |
| **Ditch H higher section** | TQ 61025 07084 | 10/08/2021 11.50 | 280ml | medium | sunny/breeze very vegetated |
| **WIDER DITCH NETWORK** | | | | | |
| **Down Sewer Main** | TQ62291 07329 | 12/08/2021 09.20 | 300ml | low, ditch recently cleared of vegetation | cloudy |
| **Down Sewer -small ditch opposite** | TQ62350 07281 | 12/08/2021 08.50 | 250ml | slight colouration, algae, floating pennywort present, medium | cloudy, warm |
| **Horse Eye Sewer** | TQ62868 07974 | 12/08/2021 10.30 | 250ml | medium, slight colouration | floating pennywort, algae, medium |
| **Rickney Sewer** | TQ62794 07932 | 12/08/2021 09.55 | 1-120ml  2-150ml | high, algae | floating pennywort dominant, cloudy |
| **Site L (Marland Sewer)** | TQ60564 07091 | 11/08/2021 12.20 | 260ml | low, some colouration | sunny |
| **GLYNLEIGH LEVEL SEWER** | | | | | |
| **Main sewer F upstream of main ditch** | TQ60549 07158 | 11/08/2021 12.20 | 400ml | low, clear | sunny |
| **Main sewer K downstream of ditch J** | TQ61166 06935 | 12/08/2021 12.00 | 300ml | low, tall reed fringe, difficult access | cloudy, overcast |
| **Main sewer I** | TQ61068 069581 | 12/08/2021 12.20 | 300ml | low, some colouration, difficult access, scrub, reed fringed | sunny intervals, warm |

**C. chinensis* specimens found, ^#^*C. chinensis* shell found

**S2 Table. Leicestershire Ditch Water Samples 2021.**

| **Sample ID** | **Collection date/time** | **What 3 words Location** | **Volume filtered** | **Sample Condition** | **Site conditions** |
| --- | --- | --- | --- | --- | --- |
| 1 | 31/01/21 | Unguarded-Grove-Decanter | 420ml | Low turbidity | Overcast, 1°C |
| 2 | 31/01/21 | Chap-City-Decanter | 90ml | Low turbidity | Overcast, 1°C |
| 3 | 31/01/21 | Cassettes-Legroom-Housing | 60ml | Low turbidity | Overcast, 1°C |
| 4 | 31/01/21 | Rattled-Games-Foresight | 60ml | Low turbidity | Overcast, 1°C |
| 5 | 31/01/21 | Observers-Rises-Stoppage | 200ml | Low turbidity | Overcast, 1°C |
| D1 | 10/01/21; 10.00 | Happily-Zealous-Mixes | 240ml | Low turbidity | Overcast, 1°C |
| D2 | 10/01/21; 10.10 | Processor-Symphonic-Winded | 240ml | Low turbidity | Overcast, 1°C |
| Top | 04/02/21; 13.05 | Repayment-Trail-Occupiers | 85ml | Low turbidity | Sunny, light breeze 6°C |
| LHS | 04/02/21: 12.55 | Mush-Arrive-Squad | 130ml | Medium turbidity | Sunny, light breeze, 6°C |
| RHS | 04/02/21: 12.50 | Smokers-Sprouted- Copiers | 65ml | Medium turbidity | Sunny, light breeze, 6°C |

**S3 Table. Stodmarsh National Nature Reserve Ditch Water samples 2020.**

| **Sample ID** | **Collection date/time** | **GPS Location** | **Volume filtered** | **Sample Condition** | **Site conditions** |
| --- | --- | --- | --- | --- | --- |
| 34 | 23/114/2020; 13.00 | 51.315018-1.192531 | 250ml | Low turbidity | Sunny, 8 degrees |
| 42 | 23/11/2020; 14.00 | 51.313210-1.193633 | 450ml | Low turbidity | Sunny, 8 degrees |
| 44 | 23/11/2020; 13.30 | 51.313603-1.193356 | 250ml | Low turbidity | Sunny, 8 degrees |
| 58 | 23/11/2020; 10.55 | 51.313686-1.196216 | 300ml | Low turbidity | Sunny, 7 degrees |
| 60 | 17/11/2020; 14.00 | 51.315713-1.197350 | 62/1: 200ml; 62/2: 150ml | Low turbidity | Sunny spells, 14 degrees |
| 62 | 17/11/2020; 14.30 | 51.314608-1.197528 | 62/1: 200ml; 62/2: 150ml | Low turbidity | Sunny spells, 14 degrees |
| 65 | 23/11/2020; 14.50 | 51.313703-1.198258 | 65/1: 70ml, 65/2: 70ml, 65/3: 70ml | High turbidity | Cloud cover, 7 degrees |
| 70 | 17/11/2020; 13.10 | 51.314726-1.197878 | 500ml | Low turbidity | Sunny, 14 degrees |
| 92 | 18/11/2020; 10.58 | 51.316634-1.201841 | 92/1: 170ml; 92/2: 150ml | Low turbidity | Sunny, breezy, warm |
| 98 | 18/11/2020; 14.00 | 51.314733-1.201146 | 98/1: 100ml; 98/2: 100ml | Medium turbidity | Windy, overcast |
| 106 | 27/11/2020; 13.00 | 51.317576-1.202240 | 400ml | Low turbidity | Overcast, 6 degrees |
| 108 | 18/11/2020; 13.00 | 51.314511-1.202374 | 108/1: 100ml; 108/2: 100ml | Medium turbidity | Clear, breezy |
| 115 | 27/11/2020; 11.45 | 51.316764-1.203014 | 220ml | Low turbidity | Overcast, 6 degrees |
| 131 | 27/11/2020; 10.30 | 51.314365-1.206790 | 500ml | Low turbidity | Overcast, 6 degrees |
| 135 | 17/11/2020; 10.20 | 51.320395-1.207171 | 300ml | Low turbidity | Sunny, 12 degrees |
| 136 | 16/11/2020; 10.55 | 51.321417-1.207495 | 136/1: 320ml; 136/2: 180ml | Low turbidity | Overcast, dry, 12 degrees |
| 146 | 16/11/2020; 13.05 | 51.320626-1.20948 | 146/1:220ml; 146/2: 200ml | Low turbidity | Overcast, dry, 12 degrees |
| 153 | 27/11/2020; 14.30 | 51.320135-1.209509 | 350ml | Low turbidity | Overcast, 7 degrees |
| 155 | 27/11/2020; 14.00 | 51.321650-1.209372 | 250ml | Low turbidity | Overcast, 6 degrees |
| 161 | 17/11/2020; 11.30 | 51.320258-1.211856 | 500ml | Low turbidity | Sunny spells, 12 degrees |

**S4 Table. Pevensey Levels Ditch Water Samples 2022.**

| **Sample ID** | **Location (what3words)** | **Collection date/time** | **Volume filtered (ml) 0.22µm/0.48µm/0.8µm** | **Turbidity of sample** | **Site conditions** |
| --- | --- | --- | --- | --- | --- |
| **Eastern Section Ditch** | | | | | |
| 1-3 pool | removers. elects. repaying | 15:15 | 160/285/1000 | Low-medium* | Breezy, sunny, warm |
| 4 | shocks. composts. dares | 15:00 | 250/400/800 | Low* | Breezy, sunny, warm |
| 5 | smiled. reference. estimate | 15:15 | 350/300/1000 | Low | Breezy, sunny, warm |
| 7 | removers. duties. exposes | 12:30 | 350/300/1000 | Low | Breezy, cloudy, warm |
| 8 | campfires. brittle. hairpin | 14:15 | 300/250/1000 | Low | Breezy, cloudy, warm |
| 8 to 18 lower side | materials. scrap. consults | 14:00 | 400/300/1000 | Low | Breezy, cloudy, warm |
| 18 lower side | caramel. restore. conspired | 14:15 | 300/300/1000 | Low | Breezy, cloudy, warm |
| 18 upper side | sprint. consented. noble | 14:15 | 400/400/1000 | Low | Breezy, cloudy, warm |
| **Western section ditch** | | | | | |
| 9 | moment. leafing. toads | 11:00 | 150/200/1000 | Low | Windy, overcast |
| Coffer to 9 | landscape. chilling. inspects | 11:00 | 300/400/1000 | Low | Windy, overcast |
| 11 opposite ditch C | loved. activity. breaches | 11:00 | 325/300/1000 | Low | Windy, overcast |
| Coffer to 11 | roadblock. grapes. scrapped | 11:00 | 250/350/1000 | Low | Windy, overcast |
| **Side ditches** | | | | | |
| Coffer to ditch G | consented. rear. strong | 16:00 | 200/200/1000 | Low* | Breezy, sunny, warm |
| Coffer to 12 | error. crackling. unhappily | 16:00 | 200/200/1000 | Low | Breezy, sunny, warm |
| Ditch H upper section | pouch. listen. gasp | 12:30 | 150/200/900 | Medium | Breezy, cloudy, warm |
| Coffer to ditch H | cascaded. thick. rice | 12:30 | 300/250/1000 | Medium | Breezy, cloudy, warm |
| Coffer to 7 | alike. blazing. occupations | 12:30 | 400/400/1000 | Low | Breezy, cloudy, warm |
| 4-J (between 4 and ditch J) | boarding. interests. annotated | 15:15 | 110/200/500 | High* | Breezy, sunny, warm |
| **Field blanks** | | | | | |
| FB1 | Next to site 8 | 13:00 | 250/250/1000 | N/A | Breezy, cloudy, warm |
| FB2 | Next to site 8 | 13:00 | 250/250/1000 | N/A | Breezy, cloudy, warm |

*indicates that there was a low water level at the sampling site.

**S5 Table. DNA information per snail specimen collected from Pevensey Levels and Stodmarsh NNR.**

| **Sample ID** | **Elution Volume** | **DNA concentration (ng/µl)** | **Snail ID from DNA** |
| --- | --- | --- | --- |
| **Pevensey Levels, Sussex** | | | |
| 1 | 200µl | 14.3 | *C. chinensis laeta* |
| 2 | 200µl | 24.1 | *C. chinensis laeta* |
|  |  |  |  |
| 4 | 200µl | 16.4 | *C. chinensis laeta* |
| 5 | 200µl | 40.6 | *C. chinensis laeta* |
| 6 | 200µl | 5.0 | *C. chinensis laeta* |
| 2b | 200µl | 27.4 | *C. chinensis laeta* |
| A | 100µl | 54.0 | ***Planorbis carinatus*** |
| B | 100µl | 59.0 | ***Sphaerium nucleus*** |
| C* | 100µl | 10.7 | ***Bithynia tentaculata*** |
| D* | 100µl | 55.0 | ***Stagnicola palustris*** |
| E* | 200µl | 17.6 | *Manacha cantiana* |
| F | 200µl | 39.0 | *C. chinensis laeta* |
| G | 200µl | 28.0 | ***Planorbis corneus*** |
|  |  |  |  |
|  |  |  |  |
| J | 100µl | 12.4 | ***Anisus vortex*** |
|  |  |  |  |
| L* | 100µl | 31.3 | ***Bithynia tentaculata*** |
| M | 100µl | 35.6 | *C. chinensis laeta* |
| N | 100µl | 118.0 | ***Sphaerium nucleus*** |
| O* | 100µl | 150.0 | *Helix aspersa* |
|  |  |  |  |
| Q | 100µl | 36.1 | ***Bithynia tentaculata*** |
|  |  |  |  |
| S* | 100µl | 54.0 | ***Sphaerium nucleus*** |
|  |  |  |  |
| U | 100µl | 45.1 | ***Bithynia tentaculata*** |
| V | 100µl | 44.6 | ***Planorbis carinatus*** |
| W | 100µl | 42.3 | ***Planorbis carinatus*** |
| **Stodmarsh NNR, Kent** | | | |
| 1* | 200µl | 4.68 | *Gyraulus crista* |
| 2* | 200µl | 2.81 | *Planorbis planorbis* |
| 3a* | 50µl | 10.6 | *Bathyomphalus contortus* |
| 4* | 200µl | 3.58 | *Hippeutis complanatus* |
| 5b* | 50µl | 38.6 | *Planorbis carinatus* |
| 6a* | 50µl | 29.7 | *Planorbarius corneus* |
| 7* | 200µl | 8.52 | *Segmentina nitida* |
| 8a* | 50µl | 33.1 | *Anisus vortex* |
| 9b* | 50µl | 30.9 | *Valvata cristata* |
| 10* | 200µl | 2.42 | *Gyraulus albus* |

* Denotes those samples used for primer/probe specificity testing. Native snails highlighted in bold were confirmed in the ditch by manual surveys (Willing 2021a [unpublished]).
