# Supplementary material for: Quantitative PCR (qPCR) assay for the specific detection of the Chinese mystery snail (Cipangopaludina chinensis) in the UK
Source: PLoS One. 2023 Oct 5;18(10):e0292163. doi: 10.1371/journal.pone.0292163 (PMC10553251; doi:10.1371/journal.pone.0292163)
Supplement: S2 File — (DOCX) [file pone.0292163.s002.docx]

# Supporting information 2

## Limit of detection and limit of quantification

Here we define LOD as the lowest standard concentration at which 95% of technical replicates amplify and LOQ is the lowest standard concentration for which the coefficient of variation (CV; equal to the standard deviation quantity divided by the mean quantity of a group of replicates) value is <35% (Klymus et al. 2019).


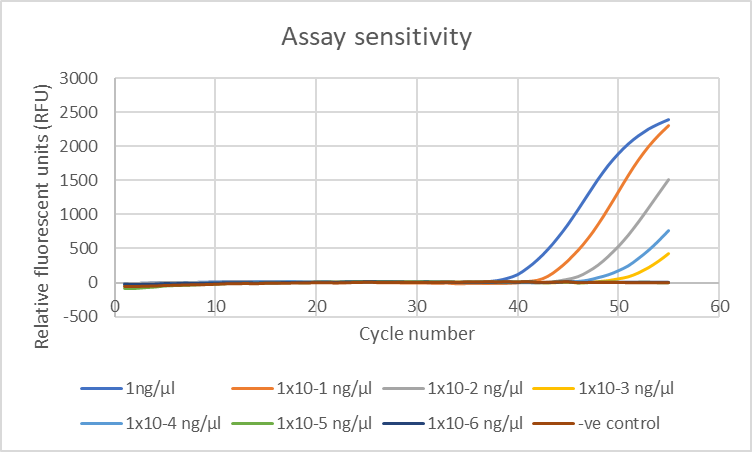


**S1 Fig. Real-time PCR result showing a seven-level standard curve for *C. chinensis* DNA.** Average values from each set of 12 replicates are shown i.e. one line per DNA dilution. Higher concentrations of DNA commence amplification before lower concentrations.

**S6 Table. PCR scores for limit of detection testing.**

| **DNA concentration** | **PCR score (out of 12)** |
| --- | --- |
| 1ng/µl | 12 |
| 1x10^-1^ ng/µl | 12 |
| 1x10^-2^ ng/µl | 12 |
| 1x10^-3^ ng/µl | 12 |
| 1x10^-4^ ng/µl^*^ | 12 |
| 1x10^-5^ ng/µl | 0 |
| 1x10^-6^ ng/µl | 0 |

^*^ DNA concentration at which 95% of technical replicates amplify, as all replicates at this DNA concentration and above amplified this is also the limit of quantification.
