# Supplementary material for: Quantitative PCR (qPCR) assay for the specific detection of the Chinese mystery snail (Cipangopaludina chinensis) in the UK
Source: PLoS One. 2023 Oct 5;18(10):e0292163. doi: 10.1371/journal.pone.0292163 (PMC10553251; doi:10.1371/journal.pone.0292163)
Supplement: Attachment — Submitted filename: PONE-D-23-17026_Reviewer_v1.pdf [file pone.0292163.s003.pdf]

PLOS ONE

qPCR assay for the specific detection of the Chinese mystery snail (Cipangopaludina chinensis) in the UK.

--Manuscript Draft--

**Manuscript Number:**	PONE-D-23-17026
**Article Type:**	Research Article
**Full Title:**	qPCR assay for the specific detection of the Chinese mystery snail (Cipangopaludina chinensis) in the UK.
**Short Title:**	eDNA detection of the Chinese mystery snail.
**Corresponding Author:**	Helen Christian Rees, Ph.D.<br>RSK ADAS Ltd<br>Helsby, UNITED KINGDOM
**Keywords:**	Ditch, environmental DNA, qPCR, Chinese mystery snail, Cipangopalaudina chinensis, non-native species, water samples, freshwater habitats.
**Abstract:**	Cipangopaludina chinensis (Gray 1833) is an East Asian freshwater snail and invasive species in many parts of the world (Global Invasive Species Database, 2022). Within the UK, it was first found at the Pevensey Levels, Sussex, and has since been reported at a second site at Southampton Common, Hampshire. Both sites are designated as Sites of Special Scientific Interest (SSSI) for their wildlife importance. Although the impacts of this species within the UK have not yet been investigated several exotic parasites of the snail have been reported and research suggests that its presence can negatively impact native snail species. This is especially important at the Pevensey Levels due to the presence of the rare freshwater mollusc Anisus vorticulus (Little Whirlpool Rams's-horn snail). Here, we have developed a Taqman based qPCR eDNA assay for the detection of C. chinensis and compared water samples tested for eDNA with results from manual survey of the ditches at the Pevensey Levels. Our eDNA analysis exhibited an overall observed percentage agreement of 80% with a kappa coefficient of agreement between manual and eDNA surveys of 0.59 (95% CI 0.31 to 0.88). Some samples determined to be negative for C. chinensis by eDNA analysis were manual survey positive, and vice versa revealing the potential for improved overall detection rates using a combination of manual and eDNA methodologies. eDNA analysis can therefore augment manual survey techniques for C. chinensis as a relatively quick and inexpensive tool for collecting presence and distribution data that could be used to inform further manual surveys and control measures within the ditches.
**Order of Authors:**	Helen Christian Rees, Ph.D.
	Gavin Measures
	Steven Kane, PhD
	Ben Maddison, PhD
**Additional Information:**	
**Question**	**Response**
**Financial Disclosure**<br><br>Enter a financial disclosure statement that describes the sources of funding for the work included in this submission. Review the submission guidelines for detailed requirements. View published research articles from *PLOS ONE* for specific examples.	This project was funded by a grant to HR from Natural England. Award reference: Chinese mystery snail, Pevensey Levels Sussex. GM of Natural England played a role in the design, sample collection, and preparation of manuscript. https://www.gov.uk/government/organisations/natural-england

Powered by Editorial Manager® and ProduXion Manager® from Aries Systems Corporation

This statement is required for submission and **will appear in the published article** if the submission is accepted. Please make sure it is accurate.<br><br>**Unfunded studies**<br>Enter: *The author(s) received no specific funding for this work.*<br><br>**Funded studies**<br>Enter a statement with the following details:<br>• Initials of the authors who received each award<br>• Grant numbers awarded to each author<br>• The full name of each funder<br>• URL of each funder website<br>• Did the sponsors or funders play any role in the study design, data collection and analysis, decision to publish, or preparation of the manuscript?<br>• **NO** - Include this sentence at the end of your statement: *The funders had no role in study design, data collection and analysis, decision to publish, or preparation of the manuscript.*<br>• **YES** - Specify the role(s) played.<br><br>* typeset	
**Competing Interests**<br><br>Use the instructions below to enter a competing interest statement for this submission. On behalf of all authors, disclose any competing interests that could be perceived to bias this work—acknowledging all financial support and any other relevant financial or non-financial competing interests.<br><br>This statement is required for submission and **will appear in the published article** if the submission is accepted. Please make sure it is accurate and that any funding sources listed in your Funding Information later in the submission form are also declared in your Financial Disclosure statement.<br><br>View published research articles from *PLOS ONE* for specific examples.	NO authors have competing interests

Powered by Editorial Manager® and ProduXion Manager® from Aries Systems Corporation

**NO authors have competing interests**<br><br>Enter: *The authors have declared that no competing interests exist.*<br><br>**Authors with competing interests**<br><br>Enter competing interest details beginning with this statement:<br><br>*I have read the journal's policy and the authors of this manuscript have the following competing interests: [insert competing interests here]*<br><br>* typeset	
**Ethics Statement**<br><br>Enter an ethics statement for this submission. This statement is required if the study involved:<br><br>• Human participants<br>• Human specimens or tissue<br>• Vertebrate animals or cephalopods<br>• Vertebrate embryos or tissues<br>• Field research<br><br>Write "N/A" if the submission does not require an ethics statement.<br><br>General guidance is provided below. Consult the submission guidelines for detailed instructions. **Make sure that all information entered here is included in the Methods section of the manuscript.**	Water samples and snail specimens were collected from several ditches at the Pevensey Levels and no institutional animal care and use committee (IACUC) or animal welfare protocol was required for their collection as generally invertebrates do not require approval for use (except cephalopods). No endangered or protected species were collected during field survey and all land was accessed with the permission of the landowners.

Powered by Editorial Manager® and ProduXion Manager® from Aries Systems Corporation

**Format for specific study types**<br><br>**Human Subject Research (involving human participants and/or tissue)**<br>• Give the name of the institutional review board or ethics committee that approved the study<br>• Include the approval number and/or a statement indicating approval of this research<br>• Indicate the form of consent obtained (written/oral) or the reason that consent was not obtained (e.g. the data were analyzed anonymously)<br><br>**Animal Research (involving vertebrate animals, embryos or tissues)**<br>• Provide the name of the Institutional Animal Care and Use Committee (IACUC) or other relevant ethics board that reviewed the study protocol, and indicate whether they approved this research or granted a formal waiver of ethical approval<br>• Include an approval number if one was obtained<br>• If the study involved *non-human primates*, add *additional details* about animal welfare and steps taken to ameliorate suffering<br>• If anesthesia, euthanasia, or any kind of animal sacrifice is part of the study, include briefly which substances and/or methods were applied<br><br>**Field Research**<br><br>Include the following details if this study involves the collection of plant, animal, or other materials from a natural setting:<br>• Field permit number<br>• Name of the institution or relevant body that granted permission	
**Data Availability**<br><br>Authors are required to make all data underlying the findings described fully available, without restriction, and from the time of publication. PLOS allows rare exceptions to address legal and ethical concerns. See the PLOS Data Policy and FAQ for detailed information.	Yes - all data are fully available without restriction

A Data Availability Statement describing where the data can be found is required at submission. Your answers to this question constitute the Data Availability Statement and **will be published in the article**, if accepted.<br><br>**Important:** Stating 'data available on request from the author' is not sufficient. If your data are only available upon request, select 'No' for the first question and explain your exceptional situation in the text box.<br><br>Do the authors confirm that all data underlying the findings described in their manuscript are fully available without restriction?	
**Describe where the data may be found in full sentences. If you are copying our sample text, replace any instances of XXX with the appropriate details.**<br><br>• If the data are **held or will be held in a public repository**, include URLs, accession numbers or DOIs. If this information will only be available after acceptance, indicate this by ticking the box below. For example: *All XXX files are available from the XXX database (accession number(s) XXX, XXX.*).<br>• If the data are all contained **within the manuscript and/or Supporting Information files**, enter the following: *All relevant data are within the manuscript and its Supporting Information files.*<br>• If neither of these applies but you are able to provide **details of access elsewhere**, with or without limitations, please do so. For example:<br><br>*Data cannot be shared publicly because of [XXX]. Data are available from the XXX Institutional Data Access / Ethics Committee (contact via XXX) for researchers who meet the criteria for access to confidential data.*<br><br>*The data underlying the results presented in the study are available from (include the name of the third party*	All the relevant data are within the manuscript and its supporting information files.

and contact information or URL).<br>• This text is appropriate if the data are owned by a third party and authors do not have permission to share the data.<br><br>* typeset	
Additional data availability information:	

Manuscript

Click here to access/download;Manuscript;C chinensis manuscript.docx

~~qPCR~~ assay for the specific detection of the Chinese mystery snail (*Cipangopaludina chinensis*) in the UK.

Short Title: eDNA detection of the Chinese mystery snail.

Helen C. Rees[1], Gavin H. Measures[2], Steven D. Kane[1], & C. Maddison[1].

[1]RSK ADAS Biotechnology, Unit 27, Technology Drive, Beeston, Nottingham, NG9 1LA.

[2]Natural England, Quay House, 2 East Station Road, Peterborough, PE2 8YY.

Corresponding Author:

Helen C. Rees, RSK ADAS Biotechnology, Unit 27, Technology Drive, Beeston, Nottingham, NG9 1LA. Email: helen.rees@adas.co.uk Telephone: +44 (0)1159 229249

Abstract

Cipangopaludina chinensis (Gray 1833) is an East Asian freshwater snail and invasive species in many parts of the world (Global Invasive Species Database, 2022). Within the UK, it was first found at the Pevensey Levels, Sussex, and has since been reported at a second site at Southampton Common, Hampshire. Both sites are designated as Sites of Special Scientific Interest (SSSI) for their wildlife importance. Although the impacts of this species within the UK have not yet been investigated several exotic parasites of the snail have been reported and research suggests that its presence can negatively impact native snail species. This is especially important at the Pevensey Levels due to the presence of the rare freshwater mollusc *Anisus vorticulus* (Little Whirlpool Rams's-horn snail). Here, we have developed a ~~Taqman based~~ qPCR eDNA assay for the detection of *C. chinensis* and compared water samples tested for eDNA with results from manual survey of the ditches at the Pevensey Levels. Our eDNA analysis exhibited an overall observed percentage agreement of 80% with a kappa coefficient of agreement between manual and eDNA surveys of 0.59 (95% CI 0.31 to 0.88). Some samples determined to be negative for *C. chinensis* by eDNA analysis were manual survey positive, and *vice versa* revealing the potential for improved overall detection rates using a combination of manual and eDNA methodologies. eDNA analysis can therefore augment manual survey techniques for *C. chinensis* as a relatively quick and inexpensive tool for collecting presence and distribution data that could be used to inform further manual surveys and control measures within the ditches.

Key Words

Ditch, environmental DNA, qPCR, Chinese mystery snail, *Cipangopalaudina chinensis*, non-native species, water samples, freshwater habitats.

Introduction

Knowledge of species distribution is critical to ecological management and conservation biology. Effective management requires the detection of populations which can sometimes be at low densities and is usually based on visual detection and counting. There has been increasing interest in using environmental DNA (eDNA) techniques to monitor freshwater invertebrate communities and in particular invasive non-native species.

eDNA describes the DNA found within an environmental sample (water, soil, sediment, air etc.) and includes DNA that originates from sloughed cellular material (e.g. skin cells); DNA that is excreted (e.g. faeces and urine); or DNA that is secreted (e.g. saliva) by organisms occupying (or visiting) the environment in question (Rees et al., 2014). The presence of an organism's DNA within a water body is short lived and eDNA has been shown to be degraded to undetectable levels within days to weeks and is affected by UV light, pH and microbial activity (Shogren et al., 2017; Seymour et al., 2018; Zulkefi et al, 2019). The detection of an organism's DNA is therefore a demonstration of its presence/recent presence and can be used instead of manual survey or capture of the organism itself (Pilliod et al., 2013; Strickler et al., 2015;).

For just over a decade eDNA has been used as a non-invasive sampling technique. It has been shown to be reliable, cost effective, less harmful to the ecosystem and correlates well with conventional survey results (Biggs et al., 2015; Darling 2019). As such, the analysis of eDNA using PCR-based approaches has become an important tool for measuring presence/absence of various aquatic species including the highly invasive mollusc *Potamopyrgus antipodarum* New Zealand mud snail within rivers (Goldberg et al, 2013; Clusa et al. 2016).

The Chinese mystery snail *Cipangopaludina chinensis* (Gray, 1833) or 'trapdoor snail' is an extremely large (40-60mm high, 30-40mm wide), globose operculate snail native to East Asia

and is the largest freshwater snail in Britain (Figure 1). The ‘trapdoor’ refers to an oval plate (operculum) which seals the aperture of the snail when the snail is fully retracted. *C. chinensis* is a problem invasive species in many parts of the world and has only relatively recently been introduced to Europe. It has been present in the Netherlands since at least 2007 (Collas et al 2017) and was found at the Pevensey Levels, Sussex, UK in 2018 (Willing 2018) and has since been reported at Southampton Common, Hampshire, UK in 2022 (Measures 2022). The species is sold for ornamental ponds and aquaria and is also eaten in certain Asian communities. It is thought that either of these may have resulted in the accidental (or intentional) introduction of *C. chinensis* at the two sites in Britain resulting in breeding populations. Females can produce up to 30 live young per year (Collas et al, 2017) and live for up to five years (Jokinen, 1982) which could produce a boom in population. Across Asia, multiple species of *C. chinensis* have been recognised and there is considerable confusion on its taxonomy. For example ~~*C.*~~ *fluminalis* is a synonym of, and ~~*C.*~~ *wingatei* is a subspecies of *C. chinensis* (Lu *et al.* 2014). Two subspecies of *C. chinensis* that are recognised are ~~*C.*~~ *chinensis chinensis* (Gray, 1833) and ~~*C.*~~ *chinensis malleata* (Reeve, 1863; also known as *C. chinensis laeta*) (Matthews *et al.* 2017)~~, the latter of which was found to be present at the Pevensey Levels, Sussex during this study~~.

The Pevensey Levels site is within an internationally important area of coastal grazing marshes which is also designated as a Special Area of Conservation and a Ramsar site. At the Pevensey Levels the snail lives and breeds in a heavily vegetated shallow drainage ditch system mostly in cattle-poached margins and ~~was~~ first found in September 2018. Survey work carried out in July 2019 found the presence of a recruiting population of the snail in an approximately 400m stretch of the ditch and further surveys carried out at this ditch system in February 2021 found a number of smaller juveniles which suggested that a breeding population was present (Willing 2021a). Little is known about the impact of *C. chinensis* in

Britain, however at the Pevensey Levels, there is a concern that should *C. chinensis* spread from its current locations within the ditch system then there will be a risk of disruption to the diverse and extensive freshwater ecosystems at the site. *C. chinensis* are considered to be an aquatic invasive species due to their ability to avoid predation and out-compete indigenous mollusc species for resources. Research has shown that their presence can negatively impact native snail species in mesocosm experiments (Johnson et al, 2009) and several exotic parasites of *C. chinensis* have been reported (Kipp et al., 2020). Additionally, they excrete a large amount of faecal matter which can affect nitrogen and phosphorous cycling in aquatic ecosystems (Olden et al. 2013). The ditch system in question is a habitat for many rare invertebrate species including rare the freshwater Mollusca the Little Whirlpool Ram's-horn Snail *Anisus vorticulus* (Troschel 1834) and the Shining ram's-horn snail *Segmentina nitida* (Muller 1774).

This study therefore set out to develop an eDNA-based assay for the detection of *C. chinensis* which could be used to inform and complement traditional manual surveys at the Pevensey Levels and the success of any eradication or mitigation operations over subsequent years. Here, we describe a qPCR assay for the detection of *C. chinensis* and demonstrate the detection of this species at this UK site.

Materials and methods

<u>Sampling Design</u>

The parts of the ditch system at the Pevensey Levels that were targeted for eDNA sampling were those where there had been previous manual records for *C. chinensis*, plus connected and nearby ditches, up to a total of 36 water samples. eDNA sampling effort aimed to collect enough samples to obtain adequate representation of occupied areas of the site.

<u>Sample collection: Pevensey Levels 2021</u>

36 ditch water samples were collected by Natural England and ADAS staff at Pevensey Levels between the 10th and 12th August 2021 (Figure 2). In addition, 32 ditch samples were collected outside the known range of *C. chinensis* (Stodmarsh, Kent and Leicestershire) and were used as assay specificity controls. These were collected by Natural England and RSK ADAS staff between the 16th January and the 27th November 2020 and 10th January and the 4th February 2021 respectively. All sample information can be found in supplemental file 1 (tables S1 to S3). Each sample was collected using its own dedicated sampling pack i.e. sterile disposable gloves, a sterile scoop, a sterile sampling bag, and a sterile 50ml syringe. 20x 30 mL water samples were collected along the length of each sampling site at the ditch system and pooled into a sterile sampling bag. Up to 500 mLs of this water was filtered through a 0.22 µm PES sterivex filter (Millipore) using a 50 mL luer-lock syringe (Table 1). After filtration of water, 95% ethanol was added as a preservative. One control field blank (distilled water) was also filtered on site.

<u>Sample Collection: Pevensey Levels 2022</u>

18 ditch water samples were collected at Pevensey Levels on the 3rd August 2022 (Figure 3). All sample information can be found in Supplemental File 1, Table S4. Two control field blanks (same volume of distilled water) were also filtered by ADAS staff next to sampling site 8.

For all samples two litres of water was collected along the length of each sampling site at the ditch system and pooled into a sterile sampling bag to allow for two types of filters to be tested for their ease of use and filterable volume of water. As in the 2021 eDNA survey a 0.22µm Sterivex polyethersulfone (PES) filter (Merck Millipore, Darmstadt, Germany) was used alongside a second filter type: 0.45µm PES filter (GVS, Stanford, North America) in combination with a 3.1µm glass fibre pre-filter (GVS, Stanford, North America) (recommended where water to be sampled is turbid - used to remove larger particles of

sediment and plant material before capturing eDNA on the main filter). Up to 500 mLs of water was filtered and 95% ethanol was added as a preservative.

DNA Extraction

Upon return to the laboratory, the preservative solution was removed from the filters and any DNA containing material captured on the filter membrane was recovered by addition of lysis buffer and proteinase K to the filter as follows:

1. For sterivex filters - 760μL of ATL buffer/proteinase K (10:1 ratio) was added to each filter.
2. For the PES/GF filter combination – 580μL ATL buffer/proteinase K (10:1) was added to each filter.

The ends of the filters were sealed and briefly agitated by vortexing before incubation overnight at 37°C. The supernatant was then extracted using the DNeasy blood and tissue kit (Qiagen) following the manufacturer's instructions with final resuspension in 200μl of elution buffer. All DNA samples were quantified using the Qubit® dsDNA BR assay kit and Qubit 3.0 Fluorometer (Invitrogen) following the manufacturer's instructions then stored at -20 °C before use. Field blanks (x1) and extraction blanks (x2) were also processed alongside the Pevensey Levels and Southampton Common sterivex filters. All eDNA extractions from filters were performed in a separate laboratory remote from snail specimen eDNA extraction and qPCR set up, using dedicated tissue extraction equipment. Disposable laboratory coats were worn, and benches and equipment were wiped down with a 10% bleach solution before and after use.

Specimen Collection and DNA extraction

Snail specimens of both *C. chinensis* and native species were collected from ditches at the Pevensey Levels site by hand netting using a survey net fitted with a 2mm mesh by Natural

England staff in August 2021 (Rees et al. 2022). Specimens were preserved in 95% ethanol prior to couriering to the RSK ADAS laboratories.

Each snail specimen was individually transferred to a clean, sterile petri dish and a photographic record made. For larger specimens a fresh sterile scalpel blade was used to remove a piece of tissue from each specimen and macerate the tissue into a fine paste before placing into a sterile 1.5 mL Eppendorf tube. After use mortar and pestles were immediately immersed in 10 % bleach for a minimum of 10 minutes and then cleaned in between samples with 10 % Distel (Tristel™), rinsed with dH_2O and then autoclaved at 121 °C for 15-20 minutes. For smaller specimens the whole organism was transferred into a sterile 1.5 mL Eppendorf tube and using a fresh sterile Eppendorf pestle was ground into a fine paste.

DNA was extracted from snail specimens using DNeasy blood and tissue kit (Qiagen) following the manufacturer's instructions: 200 µL ATL and 20 µL PK buffer was added to each specimen and incubated for 1-2 hours at 56 °C until completely digested. Final resuspension was in 100 or 200 µL AE buffer for specimens (Supplemental Information 1: Table S5). Extracted DNA was quantified using the Qubit® dsDNA BR assay kit and Qubit 3.0 fluorimeter.

Specimen Identification PCR

All PCR set up was performed in a clean 'PCR room' within a UV sterilisable cabinet within a separate laboratory to DNA extraction using dedicated equipment and PPE. To ensure the unidirectional workflow DNA extracts are collected from the DNA extraction laboratory and transferred to the PCR set-up laboratory.

DNA barcoding was performed to confirm the identity of the provided specimens using the COI primers mICOIintF 5'-GGWACWGGWTGAACWGTWTAYCCYCC-3' and jgHCO2198 5'-TAIACYTCIGGRTGICCRAARAAYCA-3' (Leray et al. 2013; Geller et al. 2013). After PCR and amplicon clean-up using the Macherey-Nagel™ NucleoSpin™ Gel

and PCR Clean-up Kit according to the manufacturers instructions, PCR products were Sanger sequenced and returned sequences identified using BLAST (Supplemental File 1: Table S6).

Species Specific Primer Design and Validation

To design primers specific to *C. chinensis* the DNA sequences for the cytochrome oxidase 1 (COI) gene for *C. chinensis* and other closely related and/or co-occurring snails commonly found at the Pevensey Levels and the wider UK were downloaded from BOLD/Genbank and their sequences aligned using BioEdit version 7.2.5. This list of species comprised the 54 species of freshwater snails known to occur in the UK, both native and non-native (Rowson et al. 2021). Primers and probes were designed using PrimerBLAST with default settings. Ten potential primer/probe combinations were generated and reduced to four using PrimerBLAST and looking for cross-species amplification.

The four potential primer/probe combinations were tested firstly on DNA extracted from *C. chinensis* followed by 16 other closely related and/or co-occurring snail species to test for cross-species reactivity. PCRs were set up in a total volume of 25 µL consisting of: 1 µL of each primer/probe (final concentrations: 0.2 µmol/L forward primer; 0.8 µmol/L reverse primer; 0.5 µmol/L probe), 12.5 µL of TaqMan® Environmental Master Mix 2.0 (containing AmpliTaq GOLD DNA polymerase), and 6.5 µL ddH2O. 3µL of DNA was added to each reaction and 12 replicates were performed per DNA sample. All controls were performed in quadruplicate with a dilution series of $1x10^{-1}$ to $1x10^{-4}$ ng/µL *C. chinensis* DNA being used as positive controls and ddH2O in place of DNA for the negative control. PCRs were run on a Bio-Rad CFX Connect real-time PCR machine as follows: an initial incubation for 5 minutes at 56.3°C then 10 minutes at 95°C; followed by 55 cycles with a melting temperature of 95°C for 30 seconds and an annealing temperature of 56.4°C for 1 minute.

Once specificity of primer/probe combinations was confirmed, the most promising primer primer/probe combination (no cross reactivity, highest RFU, best shaped curve etc.) was optimised by independently varying final primer concentrations (the probe was held at a final concentration of 0.1 µmol/L) (Wilcox *et al*, 2015). The sensitivity of the assay was tested by creating a seven-level standard curve dilution series (1ng/µl to 1x10^{-6} ng/µl). 12 replicates of each dilution were run using the optimised primer/probe concentrations to determine the standard curve slope, the limit of detection (LOD) and limit of quantification (LOQ). The optimised assay using primer/probe combination 8 (CMS 8F: 5'-GTGATTGTAACTGCTCACGCAT-3'; CMS 8R: 5'- CCCAACACCCCCTTCAACAG-3'; CMS probe: 5'- GGAGCTCCTGATATAGCTTTTCCTCGT-3', FAM/BHQ-1) was used to determine the presence/absence of *C. chinensis* within the 36 ditch samples from the Pevensey Levels and the 30 ditch samples from outside the known range of *C. chinensis* (Supplemental file 1: Table S2 and S3).

Inhibition testing

All DNA extracts were tested for inhibition by adding 3µL of extract into a qPCR for the amplification of an internal positive control (DNA derived from the plasmid pSD3 previously used in our laboratories). PCRs were set up in a total volume of 25 µL consisting of: 3 µL of internal positive control DNA (0.08 ng/µl), 1 µL of each primer/probe (final concentrations: 0.2 µmol/L forward primer; 0.4 µmol/L reverse primer; 0.1 µmol/L probe; Supplemental file 2), 12.5 µL of TaqMan® Environmental Master Mix 2.0, and 3.5 µL ddH2O. 3µL of extracted DNA template was added to each reaction and 12 replicates were performed per DNA sample. All controls were performed in quadruplicate with a dilution series of 0.08 ng/µl to 0.0032 ng/µL internal positive control DNA being used as positive controls and ddH2O in place of DNA for the negative control. PCR cycling was as above.

Statistical analysis

To measure the agreement between the two survey methods, that is, manual survey and eDNA analysis, Cohen's kappa coefficient (Cohen 1960) was calculated as follows:

$$k = \frac{\Pr(a) - \Pr(e)}{1 - \Pr(e)}$$

where Pr(a) is the relative agreement among rates, and Pr(e) is the hypothetical probability of chance agreement, using the observed data to calculate the probabilities of each method randomly giving a positive detection. If the methods are in complete agreement, then j = 1. If there is no agreement other than what would be expected by chance, j = 0. Kappa analysis did not include samples taken from outside the known range of *C. chinensis* or any other samples taken from sites that had not been manually surveyed.

Once PCR analyses had been performed, the program PRESENCE version 2.13.43 [available from http://www.mbr-pwrc.usgs.gov/software/presence.html (Mackenzie et al. 2002)] was used for occupancy modelling of the data. A single-season model was used which assumes that species are never falsely detected at a site when absent, but that may or may not be detected when present; the detection of a species at an individual site is independent of the detection of the species at all other sites; and the probability of detecting the species across all sites is constant.

Results

Detection

The limit of detection (LOD) and limit of quantification (LOQ) of primer/probe combination 8 were both found to be 1×10^{-4} ng/µL (Supplemental file 2). Here we define LOD as the lowest standard concentration at which 95% of technical replicates amplify and LOQ is the lowest standard concentration for which the coefficient of variation (CV; equal to the standard deviation quantity divided by the mean quantity of a group of replicates) value is <35% (Klymus *et al*. 2019). Detection in a water sample was indicated by at least 1 of 12 positive qPCR replicates (Biggs et al. 2015), however, all 1 of 12 results were confirmed by

retesting twice more and samples were only considered to be positive if one or both of the retests showed any positive amplifications, those that were negative on both retests were considered to be negative for *C. chinensis* DNA. It should be noted that during any subsequent analysis samples went through an additional round of freeze-thawing which can lead to DNA degradation and therefore a loss of eDNA detection. This could in part account for the sample now being negative although it should also be noted that three other samples were still positive for C. chinensis DNA when retested despite also going through an additional round of freeze-thawing. None of the samples caused inhibition of the qPCR and all the field, extraction, and PCR blanks were negative for *C. chinensis* DNA.

The optimised assay for *C. chinensis* was used on the DNA extracted from all Pevensey Levels ditch water samples and ditch samples from outside the known range of *C. chinensis*. In 2021, 16 out of the 36 Pevensey Levels samples were found to be positive for *C. chinensis* DNA (Table 1, Figure 2). When comparing the two filter types during the 2022 Pevensey Levels sampling, the 0.45 PES/GF filter combination found 6 out of the 20 samples to be positive whilst the sterivex filers only found two out of 20 samples to be positive for C. chinensis DNA (Table 2, Figure 3). All remaining samples were negative for *C. chinensis* DNA, therefore other than any ditches where *C. chinensis* was found by manual survey, *C. chinensis* is likely to be absent from these ditches (Tables 1 and 2). It was also noted that fewer live *C. chinensis* were observed during the eDNA surveys in 2022 than were observed in the eDNA surveys in 2021, although a number of empty shells were seen in 2022 at both sites.

<u>Occupancy Analysis</u>

Using the observed percentage agreement of the two methods of 0.80 (1 = 100%) and the probability of random agreement of 0.52, Cohen's kappa coefficient was calculated as 0.59

(95% CI 0.31 to 0.88) for manual survey-positive ponds versus their qPCR analysis results i.e. moderate agreement (combining all samples collected in 2021 and 2022).

Site modelling was used to calculate the occupancy estimate and the probability of detection of *C. chinensis*. Using the combined manual survey and qPCR assay results for the Pevensey Levels in each of 2021 and 2022 the occupancy estimates were calculated (Table 3). Occupancy estimates were 0.58 or 0.47 with a probability of detection of 0.10 or 0.13 respectively (Table 3).

Discussion

Assay Design

This work was undertaken to develop a species-specific qPCR assay to monitor the presence of *C. chinensis* in the UK and to compare this to manual survey data. The assay, targeting a 204bp fragment of the COI region of *C. chinensis* was developed and optimised for determining the presence of *C. chinensis* based on the detection of eDNA in ditch water samples at the Pevensey Levels, UK. eDNA-based assays require that closely related and co-occurring species whose DNA may be present in the environmental samples are considered when designing the assay to ensure their specificity (Goldberg et al, 2016). The qPCR designed in this study successfully distinguished between *C. chinensis* and other co-occurring species in that no cross-amplification was detected either in silico or in vitro. The only exception to this was *C. cathayensis* a very closely related species found to cross-amplify during in silico testing. *C. chinensis* and *C. cathayensis* are distinguishable via various anatomical characteristics (Lu et al., 2014) but have very similar COI sequences which prevented the development of species-specific primers. We were unable to source DNA from this species to test our qPCR assay in vitro, however, as the species is not present in the UK this does not affect the application of the qPCR assay. Positive amplifications were also sequenced to confirm the species identity as *C. chinensis laeta* at the site. If *C. cathayensis*

was introduced into the UK this assay may also be suitable for its detection and any positive amplifications could be confirmed by sequencing.

<u>Pevensey Levels</u>

Manual survey work carried out in July 2019 found the presence of a recruiting population of *C. chinensis* in an approximately 400m stretch of the Pevensey Levels ditch system. Further surveys carried out at this ditch system in February 2021 found a number of smaller juveniles which suggested that a breeding population was present (Willing 2021a). Confirmation of the breeding population was made during August 2021 when several *C. chinensis* specimens were collected and returned to the laboratory for use as positive control material. During tissue removal for DNA extraction, one of the specimens was found to contain juvenile snails - this species is known to give live birth during June to October potentially having more than 160 young in their lifetime (Jokinen, 1992).

In August 2021, additional manual survey work carried out following eDNA sample collection, showed that *C. chinensis* had colonised an additional 138m of ditch (downstream) since it was surveyed in February 2021 (Willing 2021b). The August 2021 eDNA survey confirmed the presence of *C. chinensis* in these areas, but also identified 5 other areas where *C. chinensis* eDNA may be present despite not being found during manual survey (Rees et al. 2022) (Figure 2). The opposite was true for four additional sites where *C. chinensis* was found by manual survey but not during eDNA survey.

Site occupancy models can be used to account for imperfect detection and were used by Schmidt et al. (Schmidt et al. 2013) to demonstrate their applicability to eDNA surveys. When applied to the Pevensey Levels data within this study, site occupancy estimates were greater than the actual observed proportion though not significantly when combining manual survey with eDNA assay. This matches the observed increase in positive detections from

15/36 to 16/36 sample sites in 2021 6/18 to 7/18 sample sites in 2022 when both techniques were combined.

Following these combined findings, three coffer dams were installed to prevent further spread of *C. chinensis* within the ditch system (Figure 3). A concern is if *C. chinensis* spreads from its original source then there is the risk of disruption to the diverse and extensive freshwater ditch ecosystems on the Pevensey Levels, a habitat for many rare invertebrate species including the rare freshwater Mollusc the Little Whirlpool Ram's-horn Snail (*Anisus vorticulus*).

The three erected coffer dams (1-3) were made of metal sheet pile construction. In April 2022, further manual searches found no live *C. chinensis* in ditch sectors lying beyond (outside) any of the coffer dams or in any of the other side channels surveyed (side ditches B, C, D, G, H and J) (Willing 2022). Therefore, Natural England commissioned a further eDNA and to confirm these results during August 2022.

Water samples taken by Natural England and RSK ADAS staff in August 2022 confirmed the continued presence of *C. chinesis* within the ditch system albeit in fewer sites than in 2021. It was also found that the 0.45µm PES filter in combination with a 3.1µm glass fibre pre-filter returned a higher number of positive qPCR results (6 samples were positive for *C. chinensis* DNA) than for those samples collected using a 0.22µm sterivex filter (2 samples were positive for *C. chinensis* DNA). Small pore size filters (0.20 to 1.5 µm) have been shown to yield the most eDNA but are prone to clogging when sampling turbid water (water quality was noted to be poor during sample collection) or where there are algal blooms (Eichmiller et al, 2016; Turner et al, 2014; Liang et al, 2013). In such conditions, either larger pore-size filter or pre-filtration can be used as was the case with the PES/GF filter combination. Pre-filtration is known to increase single species detection probability (Robson et al, 2016) and give more consistent results for community compositions. However, the use of pre-filters can

lower the DNA yield and the number of detected taxa (Majaneva et al, 2018). In the current study eDNA was extracted from both the pre-filter and the main filter to minimise any DNA loses (Bruce et al. 2021). A larger pore size filter allows an increased volume of water to be filtered but there is a trade-off in that the smallest particles containing eDNA may pass through the filter. In this case similar volumes of water were filterable through the Sterivex and PES/GF filters.

C. chinensis DNA was not found in or around the coffer dam erected between sites 9 and 11 but was found within side ditch H – upper section. Although the addition of the three coffer dams could partly explain this for the side ditches (except H which was still positive for C. chinensis DNA), the water quality was poor and there had been a period of very hot weather prior to the 2022 sampling. This caused water levels to drop considerably compared with 2021 levels leaving significant areas of exposed mud at all sampled sites where lots of *C. chinensis* shells were found. It is known that *C. chinensis* favour marginal areas during the summer months, after which the snails migrate to deeper waters for the winter months (favouring the deeper more central areas) (Collas et al 2017, Jokinen, 1982). This suggests that the *C. chinensis* population could have been reduced compared with previous years thus reducing the amount of *C. chinensis* DNA present. The concentration of eDNA in a water body will depend on the rate of production versus how long it persists in the environment (Dejean et al. 2011). The rate of eDNA production for a species will depend on many factors including: the number of individuals present, their physiology and metabolism; and temperature (Treguier et al. 2014). eDNA degradation is likely to have degraded at a faster rate due to the hot weather and the high UV levels associated with this hot weather (Dejean et al. 2011; Treguier et al. 2014; Barnes et al. 2014; Pilliod et al. 2014) which could help to explain why sites which were positive for *C. chinensis* in 2021 were negative for *C. chinensis* in 2022.

During the 2021 surveys it was discovered that a new shallow water scrape had recently been created in a field lying on the far western side at the outflow end of the infested ditch (Figure 2). The scrape was not surveyed due to access issues and with no apparent ditch connection with the infested ditch colonisation by *C. chinensis* seems unlikely, however if the snail was accidentally (or intentionally) introduced, the shallow water conditions are likely to suit it.

Conclusions

This study has shown that eDNA sampling combined with manual surveys has allowed Natural England to determine the spread of *C. chinenis* in the ditch network at the Pevensey Levels. The results gave confidence that the placement of the three coffer dams were in the right location and a fourth coffer dam, planned for 2023, is to be placed in side ditch J. The implications of the long-term use of coffer dams will need to be considered as there is a concern over the possibility of the site flooding in the winter and whether water levels can be managed long-term. Natural England are planning to dredge the ditch over the next few years and eDNA sampling will be invaluable in determining the success of this operation especially if the snails are in low numbers. The new shallow scrape that was found to be suitable for colonisation by *C. chinensis* (Willing 2022) should also be surveyed by both manual and eDNA survey.

List of Abbreviations

Cytochrome oxidase I (COI), Environmental DNA (eDNA), quantitative polymerase chain reaction (qPCR), National Nature Reserve (NNR), Site of Special Scientific Interest (SSSI).

Competing interests

None

Data Accessibility Statement

All data can be found within the main manuscript or supporting files.

Authors contributions

HR and GM conceived and designed the experiments; SK performed the experiments; HR and BM analysed the data and prepared the figures; GM facilitated provision of the samples from the surveyor; HR wrote the manuscript; all authors reviewed the manuscript.

Acknowledgements

We would like to thank the Natural England Area Team staff for assisting in collection of the water samples and Dr Martin Willing (conchologist) for *C. chinensis* survey confirming the spread of the species further downstream in August 2021.

References

Barnes, M.A., Turner, C.R., Jerde, C.L., Renshaw, M.A., Chadderton, W.L., Lodge, D.M. (2014). Environmental conditions influence eDNA persistence in aquatic systems. Environmental Science and Technology, 48, 1819–1827. https://doi.org/10.1021/es404734p

Biggs, J., Ewald, N., Valentini, A., Gaboriaud, C., Dejean, T., Griffiths, R. A., Foster, J., Wilkinson, J. W., Arnell, A., Brotherton, P., Williams, P. & Dunn, F. (2015). Using eDNA to develop a national citizen science-based monitoring programme for the great crested newt (*Triturus cristatus*). Biological Conservation, 183**,** 19-28.

Bruce, K., Blackman, R.C., Bourlat., Hellstrom, M., Bakker, J., …..Deiner, K. (2021). A Practical guide to DNA-based methods for biodiversity assessment. Pensoft, Sofia, Bulgaria.

Clusa, L., Ardura, A., Gower, F., Miralles, L., Tsartsianidou, V., Zaiko, A., Garcia-Vazquez, E. (2016). An Easy Phylogenetically Informative Method to Trace the Globally Invasive Potamopyrgus Mud Snail from River's eDNA. PLoS ONE 11(10): e0162899. https://doi.org/10.1371/journal.pone.0162899

Cohen, J. (1960). A coefficient of agreement for nominal scales. Educational and Psychological Measurement. 20 (1): 37–46. https://doi.org/10.1177/001316446002000104

Collas, F.P.L. Breedveld, S.K.D., Matthews, J., Can Der Velde, G., Leuven, R.S.E.W. (2017). Invasion biology and risk assessment of the recently introduced Chinese mystery snail, *Bellamya (Cipangopaludina) chinensis* (Gray, 1834), in the Rhine and Meuse River basins in Western Europe. Aquatic Invasions 12: 275-286. https://doi.org/10.3391/ai.2017.12.3.02

Darling, J.A. (2019). How to learn to stop worrying and love environmental DNA monitoring. Aquatic Ecosystem Health & Management 22: 440–451, https://doi.org/10.1080/14634988.2019.1682912

Dejean, T., Valentini, A., Duparc, A., Pellier-Cuit, S., Pompanon, F., Taberlet, P., MIAUD, C. (2011). Persistence of Environmental DNA in Freshwater Ecosystems. PLoS ONE 6(8): e23398. https://doi.org/10.1371/journal.pone.0023398

Eichmiller, J.J., Miller, L.M., Sorensen, P.W. (2016). Optimizing techniques to capture and extract environmental DNA for detection and quantification of fish. Molecular Ecology Resources 16: 56-68. https://doi.org/10.1111/1755-0998.12421

GLOBAL INVASIVE SPECIES DATABASE, (2021). Species profile: *Cipangopaludina chinensis*. Downloaded from http://www.iucngisd.org/gisd/species.php?sc=1812 on 06-12-2021.

Geller, J., Meyer, C., Parker, M., Hawk, H. (2013). Redesign of PCR primers for mitochondrial cytochrome c oxidase subunit I for marine invertebrates and application in all-taxa biotic surveys. Molecular Ecology Resources, 13, 851-861. https://doi.org/10.1111/1755-0998.12138

Goldberg, C.S., Sepulveda, A., Ray, A., Baumgardt, J., Waits, L.P. (2013). Environmental DNA as a new method for early detection of New Zealand mudsnails (*Potamopyrgus antipodarum*). Freshwater Science, 32: 792-800. https://doi.org/10.1899/13-046.1

Gray, J. E. (1834). *Paludina chinensis*. Pg 599 and Plate IV in Cuvier's Animal Kingdom, Volume 12, Mollusca and Radiata (E. Griffith & E. Pidgeon, eds.) Whittaker & Co, London.

Goldberg, C.S., Turner, C.R., Deiner, K., Klymus, K.E., Thomsen, P.F., Murphy, M.A., Spear, S.F., McKee, A., Oyler-McCance, S.J., Cornman, R.S., Laramie, M.B., Mahon, A.R., Lance, R.F., Pilliod, D.S., Strickler, K.M., Waits, L.P., Fremier, A.K., Takahara, T., Herder, J.E., Taberlet, P. (2016). Critical considerations for the application of environmental DNA methods to detect aquatic species. Methods in Ecology and Evolution 7: 1299–1307. https://doi.org/10.1111/2041-210x.12595

Johnson, P.T.J., Olden, J.D., Solo0mon, C.T., Zanden, M.J.V. (2009). Interactions among invaders: community and ecosystem effects of multiple invasive species in an experimental aquatic system. Oecologia, 159: 161-170. https://doi.org/10.1007/s00442-008-1176-x

Jokinen, E. H. 1982. *Cipangopaludina chinensis* (Gastropoda: Viviparidae) in North America, review and update. Nautilus 96(3):89-95.

Jokinen, E. H. 1992. The freshwater snails (Mollusca: Gastropoda) of New York State. The University of the State of New York, The State Education Department, The New York State Museum, Albany, New York.

Kipp, R.M., Benson, A.J., Larson, J., Fusaro, A., Morningstar, C. (2020). *Cipangopaludina chinensis* (Gary, 1934): U.S. Geological Survey, Nonindigenous Aquatic Species Database, Gainesville, FL, and NOAA Great Lakes Aquatic Nonindigenous Species Information System, Ann Arbor, Michigan, USA. [Accessed 06/01/2023]

Klymus, K.E., Merkes, C.M., Allison, M.J., Goldberg, C.S., Helbing, C.C., Hunter, M.E., Jackson, C.A., Lance, R.F., Mangan, A.M., Monroe, E.M., Piaggio, A.J., Stokdyk, J.P., Wilson, C.C., Richter, C.A. (2020). Reporting the limits of detection and quantification for

environmental DNA assays. Environmental DNA 2: 271–282. https://doi.org/10.1002/edn3.29

Leray, M., Yang, J.Y., Meyer, C.P., Mills, S.C., Agudelo, N., Ranwez, V., Boehm, J.T., Machida, R.J. (2013). A new versatile primer set targeting a short fragment of the mitochondrial COI region for metabarcoding metazoan diversity: application for characterizing coral reef fish gut contents. Frontiers in Zoology, 10, 34. https://doi.org/10.1186/1742-9994-10-34

Liang, Z., Keeley, A. (2014). Filtration recovery of extracellular DNA from environmental water samples. Environmental Science and Technology, 47, 9324-9331. https://doi.org/10.1021/es401342b

Lu, H-F., Du, L-N., Li, Z-Q., Chen, X-Y., Yang, J-X. (2014). Morphological analysis of the Chinese Cipangopaludina species (Gastropoda; Caenogastropoda: Viviparidae). Zoological Research, 35 (6): 510-527. https://doi.org/10.13918/j.issn.2095-8137.2014.6.510

MacKenzie, D. I., Nichols, J. D., Lachman, G. B., Droege, S., Royle J. A., Langtimm, C. A. (2002). Estimating site occupancy rates when detection probabilities are less than one. Ecology 83(8): 2248-2255

Majaneva, M., Diserud, O.H., Eagle, S.H.C., Bostrom, E., Hajibabaei, M., Ekrem, T. (2018). Environmental DNA filtration techniques affect recovered biodiversity. Scientific Reports, 8, 4682. https://doi.org/10.1038/s41598-018-23052-8

Matthews, J., Collas, F.P.L., De Hoop, L. Van Der Velde, G., Leuven, R.S.E.W. (2017). Risk assessment of the alien Chinese mystery snail (*Bellamya chinensis).* Series of Reports Environmental Science, Institute for Water and Wetland Research, Radboud University.

Measures, G.H., (2022). A survey to assess the presence of the Chinese Mystery Snail (*Cipangopaludina chinensis*) at Southampton Common SSSI - 24th – 25th August 2022. Natural England (unpublished report).

Molluscabase (2021). MolluscaBase. *Cipangopaludina chinensis* (Gray in Griffith and Pidgeon, 1833). Accessed at: http://www.molluscabase.org/aphia.php?p=taxdetails&id=594807 on 2021-12-06.

Müller, O. F. (1774). Vermium terrestrium et fluviatilium, seu animalium infusorium, Helminthicorum, et testaceorum, non marinorum, succincta historia. vol 2: I-XXXVI, 1-214.

Olden, J.D., Ray, L., Mimis, M.C., and Horner-Devine, M.C. (2013). Filtration rates of the non-native Chinese mystery snail (*Bellamya chinensis*) and potential impacts on microbial communities. Limnetica, 32(1): 107–120. ISSN: 0213-8409.

Pilliod, D.S., Goldberg, C.S., Arkle, R.S., Waits, L.P. (2013). Estimating occupancy and abundance of stream amphibians using environmental DNA from filtered water samples. Canadian Journal of Fisheries and Aquatic Sciences 70: 1123-1130, https://doi.org/10.1139/cjfas-2013-0047

Rees, H. C., Maddison, B. C., Middleditch, D. J., Patmore, J. R. M. & Gough, K.C. (2014). The detection of aquatic animal species using environmental DNA – a review of eDNA as a survey tool in ecology. Journal of Applied Ecology, 51:1450-1459 https://doi.org/10.1111/1365-2664.12306

Rees, H.C., Measures, G.H., Kane, S.D., Maddison, B.M. (2022). Developing eDNA techniques for the detection of *Cipangopaludina chinensis* Chinese mystery snail in ditch systems at Pevensey Levels, Sussex, 2021. Natural England Commissioned Reports, NECR410.

http://publications.naturalengland.org.uk/publication/5446315801837568?cache=1673453508.26

Reeve, L. A. (1863). Monograph of the genus Paludina. Conchologica Iconica Volume 14 (1864), unpaginated. Lovell Reeve & Co, London.

Robson, H. L. A., Noble, T.H., Saunders, R.J., Robson, S.K.A., Burrows, D.W., Jerry, D.R. (2016). Fine-tuning for the tropics: application of eDNA technology for invasive fish detection in tropical freshwater ecosystems. Molecular Ecology Resources 16, 922–932. https://doi.org/10.1111/1755-0998.12505

Rowson, B., Powell, H., Willing, M., Dobson., M., Shaw, H. (2021). Freshwater Snails of Britain and Ireland. Telford: FSC Publications.

Seymour, M., Durance, I., Cosby, B.J., Ransom-Jones, E., Deiner, K., Ormerod, S.J., Colbourne, J.K., Wilgar, G., Carvalho, G.R., de Bruyn, M., Edwards, F., Emmett, B.A., Bik, H.M., Creer, S. (2018). Acidity promotes degradation of multi-species environmental DNA in lotic mesocosms. Communications Biology 1: 4. https://doi.org/10.1038/s42003-017-0005-3

Shogren, A.J., Tank, J.L., Andruszkiewicz, E., Olds, B., Mahon, A.R., Jerde, C.L., Bolster, D. (2017). Controls on eDNA movement in streams: Transport, Retention, and Resuspension. Scientific Reports 7: 5065, https://doi.org/10.1038/s41598-017-05223-1

Schmidt, B.R., Kery, M., Ursenbacher, S., Hyman, O.J., Collins, J.P. (2013). Site occupancy models in the analysis of env a ironmental DNA presence/absence surveys: a case study of an emerging amphibian pathogen. Methods in Ecology and Evolution, 4: 646-653. https://doi.org/10.1111/2041-210X.12052

Strickler, K.M., Fremier, A.K., Goldberg, C.S. (2015). Quantifying effects of UV-B, temperature, and pH on eDNA degradation in aquatic microcosms. Biological Conservation 183: 85–92. https://doi.org/10.1016/j. biocon.2014.11.038

Treguier, A., Paillisson, J.M., Dejean, T., Valentini, A., Schlaepfer, M.A., Roussel, J.M. (2014). Environmental DNA surveillance for invertebrate species: advantages and technical limitations to detect invasive crayfish *Procambarus clarkii* in freshwater ponds. Journal of Applied Ecology. 51, 871–879. https://doi.org/10.1111/1365-2664.12262

Troschel, F. H. (1834). De Limnaeaceis seu de Gastropodis pulmonatis quae nostris in aquis vivunt. Berolini, G. Eichler.

Turner, C.R., Barnes, M.A., Xu, C.C.Y., Jones, S.E., Jerde, C.L., Lodge, D.M. (2015). Particle size distribution and optimal capture of aqueous microbial eDNA. Methods in Ecology and Evolution, 5, 676-684. https://doi.org/10.1111/2041-210X.12206

Unstad, K.M., Uden, D.R., Allen, C.R., Chaine, N.M., Haak, D.M., Kill, R.A., Pope, K.L., Stephen, B.J., Wong, A. (2013). Survival and behavior of Chinese mystery snails (*Bellamya chinensis*) in response to simulated water body drawdowns and extended air exposure.

Management of Biological Invasions: 4 (2): 123–127. Thehttp://dx.doi.org/10.3391/mbi.2013.4.2.04

Willing, M., (2021a). A survey to assess the status of the Chinese Mystery Snail *Cipangopaludina chinensis* & the Little Whirlpool Ram's-horn snail *Anisus vorticulus* in a ditch and it's connecting channels on Glynleigh Level, Pevensey Levels (February – March 2021). Report to Natural England (unpublished).

Willing, M. (2021b). Appendix Notes to accompany: A survey to assess the status of the Chinese Mystery Snail *Cipangopaludina chinensis* & the Little Whirlpool Ram's-horn snail *Anisus vorticulus* in a ditch & it's connecting channels on Glynleigh Level, Pevensey Levels (February – March 2021). August 2021. Report to Natural England (unpublished).

Willing, M. (2022). A survey to assess the presence of the Chinese Mystery Snail *Cipangopaludina chinensis* in relation to the recent placement of three coffer dams and resurvey of a site supporting the Little Whirlpool Ram's-horn snail *Anisus vorticulus* on Glynleigh Level, Pevensey Levels, April 2022. Report to Natural England (unpublished).

Zulkefli, N.S., Kim, K-H., Hwang, S-J. (2019). Effects of Microbial Activity and Environmental Parameters on the Degradation of Extracellular Environmental DNA from a Eutrophic Lake. International journal of Environmental Research and Public Health 16. https://doi.org/10.3390/ijerph16183339

Table 1. Summary of *C. chinensis* survey and PCR status of the 36 water samples collected from the Pevensey Levels in 2021.

Sampling Site	Volume filtered (ml)	DNA (ng/µL)	qPCR results (2021)	Manual Survey result (2021)
**EASTERN SECTION DITCH**				
1-3 pool - 1	230	3.71	0/12	Not found
1-3 pool - 2	150; 150[*]	0.88	0/12	Not found
4	130	2.74	0/12	Not found
5	250	2.78	4/12	Not found
6	260	4.68	11/12	Not found
6-7	400	4.12	11/12	Present
7	100; 100[*]	5.74	12/12	Present
8	500	5.29	0/12	Present
8-18 lower side	500	13.50	12/12	Present
8-18 upper side	200	21.40	12/12	Present
18	300	2.88	12/12	Present
18-7 lower	400	13.30	12/12	Present
18-7 upper	250	23.30	1/12, 3/12, 4/12[#]	Present
**WESTERN SECTION DITCH**				
12	120; 100[*]	4.61	11/12	Present
17-16	170; 170[*]	21.80	0/12	Present
16	150; 150[*]	11.20	0/12	Present
End of barn at oak tree (near 16)	150	4.66	1/12, 0/12, 2/12[#]	Present
15	90; 80[*]	8.88	0/12	Present
11 opposite ditch C	250	4.48	1/12, 3/12, 2/12[#]	Not found
9 opposite ditch D	300	3.01	1/12, 4/12, 4/12[#]	Not found
13 opposite scrape in field	140; 140[*]	10.90	0/12	Not found
**SIDE DITCHES**				
4-J (between 4 and ditch J)	350	2.77	0/12	Not found
Ditch J	265	1.49	0/12	Not found
Ditch A	230	4.95	0/12	Not found
Ditch G lower (near 12)	70; 80[*]	5.41	2/12, 4/12, 5/12[#]	Present
Ditch G upper above culvert	290	7.08	0/12	Not found
Ditch H lower section (near 7)	250	2.18	4/12	Present
Ditch H higher section	280	15.60	2/12, 2/12, 3/12[#]	Not found
**WIDER DITCH NETWORK**				
Down Sewer Main	300	1.37	0/12	N/S
Down Sewer - small ditch opposite	250	5.41	0/12	N/S
Horse Eye Sewer	250	2.11	0/12	N/S
Rickney Sewer	120; 150[*]	2.54	0/12	N/S
Site L (Marland Sewer)	260	2.89	0/12	N/S
**GLYNLEIGH LEVEL SEWER**				

Sampling Site	Volume filtered (ml)	DNA (ng/μL)	qPCR results (2021)	Manual Survey result (2021)
Main sewer F upstream of main ditch	400	1.02	0/12	Not found
Main sewer K downstream of ditch J	300	2.01	0/12	Not found
Main sewer I	300	3.58	0/12	Not found

Green highlighted samples were found to be positive for C. chinensis by both qPCR and manual survey; orange highlighted surveys were found to be positive for C. chinensis by qPCR but not manual survey; blue highlighted samples were found to be positive for C. chinensis by manual survey but not by qPCR.

N/S denotes those sites where manual survey did not take place.

* *Samples were more than one sterivex filter had to be used due to filter clogging.*

qPCR was repeated for these samples to confirm positivity.

Table 2. Summary of *C. chinensis* survey and PCR status of the water samples collected from the Pevensey Levels in 2022.

**Sampling site**	**Volume filtered (mL)**		**DNA (ng/µL)**		**qPCR result (2022)**		**Manual survey result (2021/2022)**
	**0.22µm**	**0.45µm**	**0.22µm**	**0.45µm**	**0.22µm**	**0.45µm**	
**EASTERN SECTION DITCH**							
1-3 pool	160	285	30.10	40.90	0/12	0/12	Not found
4	250	400	8.54	7.04	0/12	0/12	Not found
5	350	300	14.80	14.50	0/12	0/12	Not found
6	350	300	8.49	16.30	0/12	4/12	Present (juvenile)
8	300	250	2.40	3.08	5/12	9/12	Present
8 to 18 lower side	400	300	8.91	2.67	0/12	1/12, 2/12, 0/12[#]	Present
18 lower side	300	300	9.64	12.40	0/12	11/12	Present
18 upper side	400	400	17.80	15.70	0/12	0/12	Present
**WESTERN SECTION DITCH**							
9	If 150	200	11.60	5.19	0/12	0/12	Not found
Coffer to 9	300	400	32.10	55.00	0/12	1/12, 0/12, 0/12[#*]	Not found
11 opposite ditch C	325	300	38.50	35.00	0/12	0/12	Not found
Coffer to 11	250	350	14.10	32.90	0/12	0/12	Not found
**SIDE DITCHES**							
Coffer to ditch G	200	200	27.10	28.30	0/12	0/12	Not found
Coffer to 12	200	200	20.10	27.00	0/12	0/12	Present
Ditch H upper section	150	200	12.10	0.848	4/12	1/12, 1/12, 1/12[#]	Not found[¥]
Coffer to ditch H	300	250	54.00	9.40	0/12	0/12	Not found (operculum)[≠]
Coffer to 7	200	200	32.40	8.54	1/12, 0/12, 2/12[#]	12/12	Present
4-J (between 4 and ditch J)	200	200	20.90	7.92	1/12, 0/12, 0/12[#*]	0/12	Not found

Green highlighted samples were found to be positive for C. chinensis by both qPCR and manual survey; orange highlighted surveys were found to be positive for C. chinensis by qPCR but not manual survey; blue highlighted samples were found to be positive for C. chinensis by manual survey but not by qPCR. 0.22µm refers to the Sterivex filter and 0.45µm refers to the PES-GF filter combination.

[#] *qPCR was repeated for these samples to confirm positivity.*

[*] *Samples found to be negative on both retests and are therefore assumed to be negative.*

[≠] in April 2022, the only evidence of C. chinensis presence was the finding of a snail operculum which may originate from by before the ditch was dammed.

[¥]this site was heavily over-shaded by brambles and have no obvious drainage towards the Glynleigh sewer. No snails were found in April 2022.

Table 3. Parameter estimates for a combination of manual survey and qPCR methods using a single-season model Ψ(-), p(-),that is, assuming constant occupancy and detection.

Model	N	-2 log likelihood	Ψ (95% CRI)	Est. P (95% CRI)	SE (P)
Manual survey plus qPCR (Pevensey Levels 2021)	2	79.36	0.58 (0.38, 0.76)	04874 (0.52, 0.88)	0.10
Manual survey plus qPCR (Pevensey Levels 2022)	2	39.47	0.47 (0.25, 0.71)	0.77 (0.44, 0.93)	0.13

Where N = number of parameters, Ψ = occupancy estimate, P = estimated detection rate. Sample size = 36 sites (Pevensey Levels 2021), 18 sites (Pevensey Levels 2022).

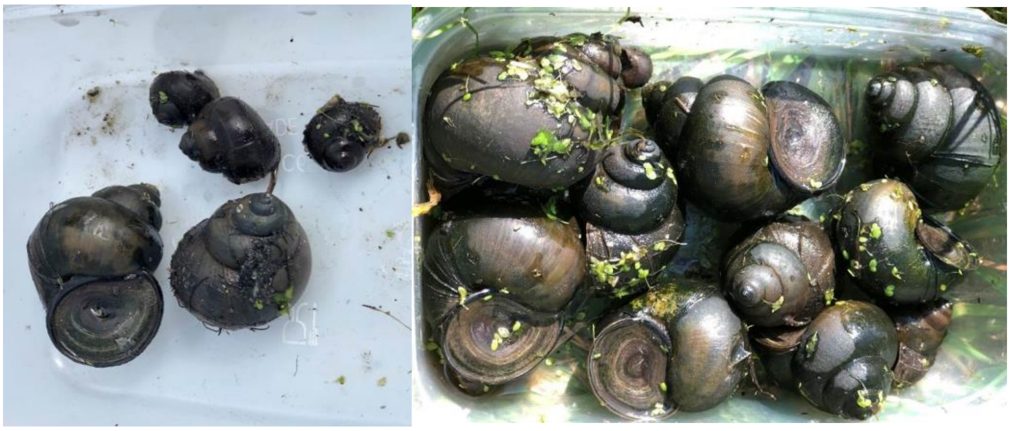

Figure 1. Images of *C. chinensis. S*pecimens collected from Pevensey Levels August 2021 (© Gavin Measures, NE).

Figure 2. Ditch system at Pevensey Levels showing C. chinensis DNA results, August 2021

Blue diamonds and orange stars mark the eDNA sampling sites and whether positive or negative for C. chinensis DNA (respectively). Black arrows show direction of flow of main ditch. Side ditches are labelled A-J.

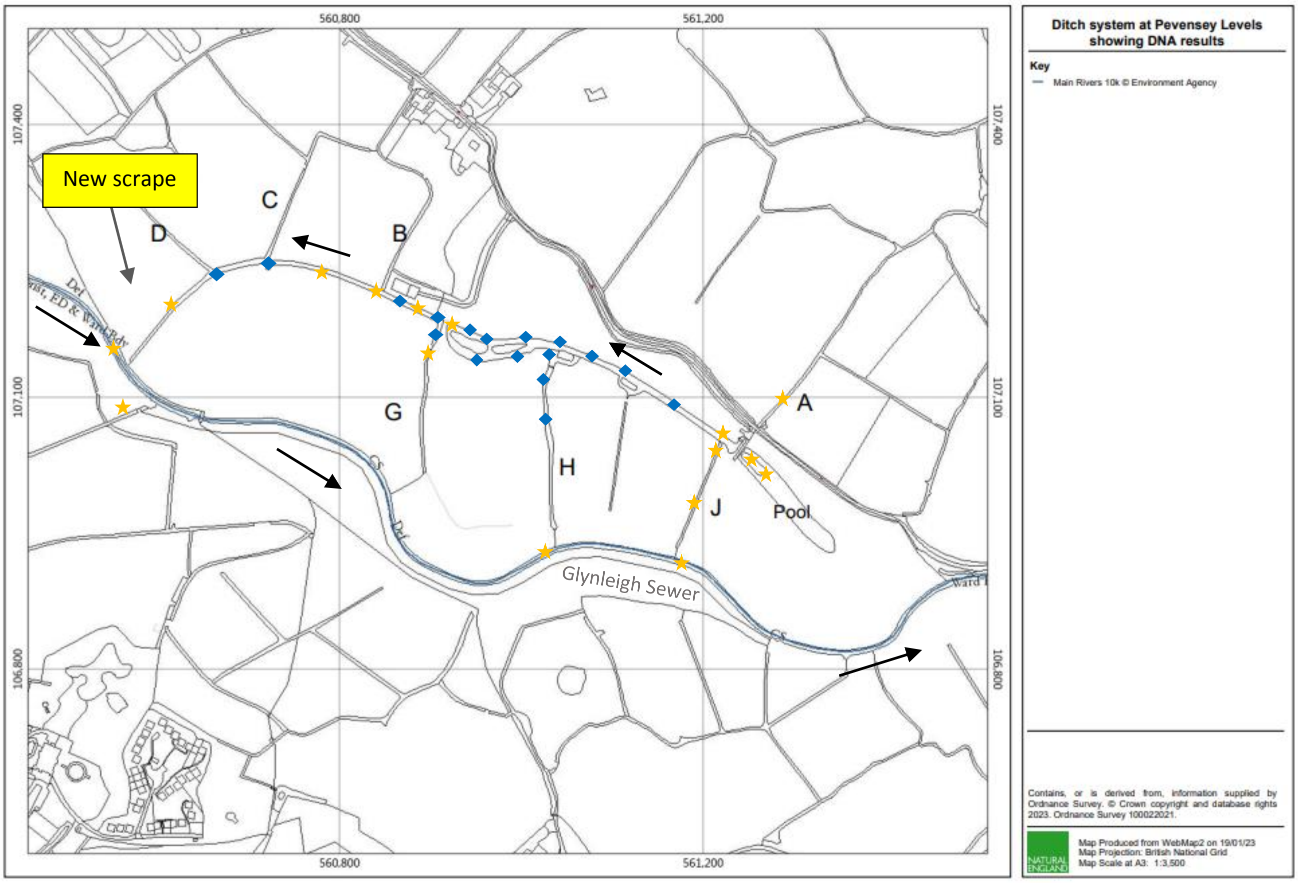

 Click here to access/download;Figure;Figure 3.docx

Figure 3. Ditch system at Pevensey Levels showing C. chinensis DNA results, August 2022

Blue diamonds and orange stars mark the eDNA sampling sites and whether positive or negative for C. chinensis DNA (respectively). Side ditches are labelled A-J. Black lines indicate the sites of the three coffer dams installed in 2021/2022 (numbered 1 to 3 from left to right).

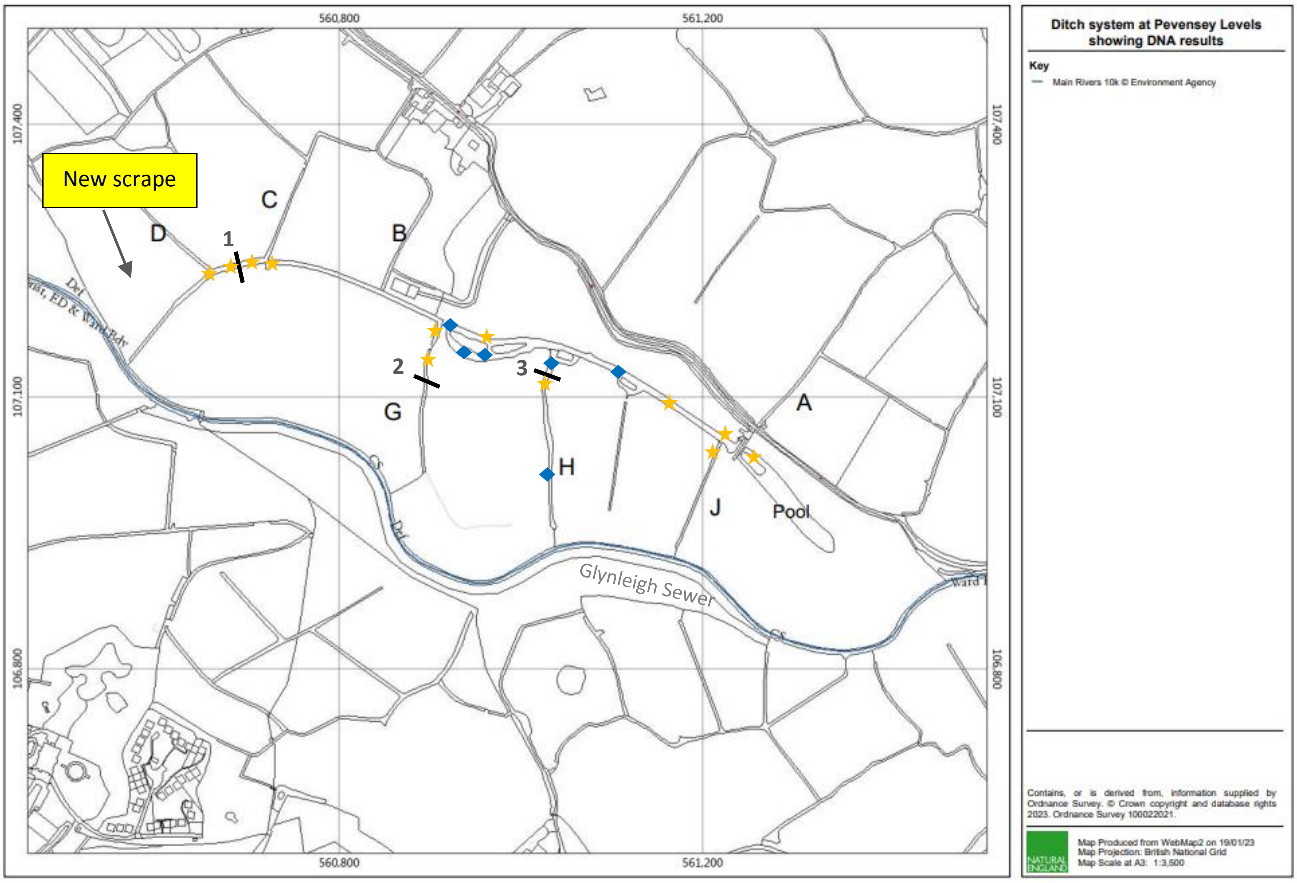

Click here to access/download
Supporting Information
Supplemental File 1.docx

Click here to access/download
Supporting Information
Supplemental file 2.docx
