# Supplementary material for: Quantitative PCR (qPCR) assay for the specific detection of the Chinese mystery snail (Cipangopaludina chinensis) in the UK
Source: PLoS One. 2023 Oct 5;18(10):e0292163. doi: 10.1371/journal.pone.0292163 (PMC10553251; doi:10.1371/journal.pone.0292163)
Supplement: Attachment — Submitted filename: Response to reviewers.docx [file pone.0292163.s004.docx]

**Academic Editor: Line numbers refer to the original manuscript.**

The Reviewers presented very detailed reports, which I fell that will contribute a lot to improvement of the final version of the MS. Please, take special attention of Reviewer 2 on the sequencing the samples of eDNA. This is important to support the specificity of the proposed assay.

*Please see specific comments to reviewer 1 and 2.*

*Other editing as per style requirements have also been made.*

*A few changes have been made to the reference list as follows:*

1. *Four references removed and stated in body of text as required by journal*
   a. *Measures, G.H., (2022). A survey to assess the presence of the Chinese Mystery Snail (Cipangopaludina chinensis) at Southampton Common SSSI - 24th – 25th August 2022. Natural England (unpublished report).*
   b. *Willing, M., (2021a). A survey to assess the status of the Chinese Mystery Snail Cipangopaludina chinensis & the Little Whirlpool Ram’s-horn snail Anisus vorticulus in a ditch and it’s connecting channels on Glynleigh Level, Pevensey Levels (February – March 2021). Report to Natural England (unpublished).*
   c. *Willing, M. (2021b). Appendix Notes to accompany: A survey to assess the status of the Chinese Mystery Snail Cipangopaludina chinensis & the Little Whirlpool Ram’s-horn snail Anisus vorticulus in a ditch & it’s connecting channels on Glynleigh Level, Pevensey Levels (February – March 2021). August 2021. Report to Natural England (unpublished).*
   d. *Willing, M. (2022). A survey to assess the presence of the Chinese Mystery Snail Cipangopaludina chinensis in relation to the recent placement of three coffer dams and resurvey of a site supporting the Little Whirlpool Ram’s-horn snail Anisus vorticulus on Glynleigh Level, Pevensey Levels, April 2022. Report to Natural England (unpublished).*
2. *Three references removed as not actually referenced in the text*
   a. *GLOBAL INVASIVE SPECIES DATABASE, (2021). Species profile: Cipangopaludina chinensis. Downloaded from http://www.iucngisd.org/gisd/species.php?sc=1812 on 06-12-2021.*
   b. *Molluscabase (2021). MolluscaBase. Cipangopaludina chinensis (Gray in Griffith and Pidgeon, 1833). Accessed at: http://www.molluscabase.org/aphia.php?p=taxdetails&id=594807 on 2021-12-06.*
   c. *Unstad, K.M., Uden, D.R., Allen, C.R., Chaine, N.M., Haak, D.M., Kill, R.A., Pope, K.L., Stephen, B.J., Wong, A. (2013). Survival and behavior of Chinese mystery snails (Bellamya chinensis) in response to simulated water body drawdowns and extended air exposure. Management of Biological Invasions: 4 (2): 123–127. Thehttp://dx.doi.org/10.3391/mbi.2013.4.2.04*

*Figure 2 and 3 have been amended in line with the journals copyright policy and also reviewer 2s additional notes.*

*There were some minor occurrences of overlapping text with one of our previous publications therefore minor editing has been carried out in the introduction lines 37-57 where we felt this issue occurred. We are unsure as to what other changes can be made as there are only so many was to introduce the topic eDNA. Should a further edit be required by the journal then we will endeavour to make further changes.*

**Reviewer 1: Line numbers refer to original manuscript.**

Line 1: qPCR has been changed to Quantitative PCR (qPCR) as requested.

Line 22: Taqman based has been removed and text changed to ‘we have developed a qPCR-based’ as requested.

Line 26-28: Sentence rewritten for clarity as requested. ‘Some samples which were qPCR negative for C. chinensis were positive by manual survey, and vice versa revealing the potential for improved overall detection rates when using a combination of manual and eDNA methodologies.’

Line 45: Additional references have been added.

- Lydolph et al. 2005
- Haile et al. 2009

Line 56: Grey 1833 has been added as requested.

Line 72-75: *Cipangopaludina fluminalis* (Heude, 1890) replaces *C. fluminalis; Cipangopaludina wingatei* (Smith, 2000) replaces *C. wingatei; Cipangopaludina chinensis chinensis* replaces *C. chinensis chinensis*; and *Cipangopaludina chnensis laeta* replaces *C. chinensis laeta* as requested.

Line 75-76: The following text has been deleted as requested. ‘the latter of which was found to be present at the Pevensey Levels, Sussex during this study.’

Line 80: ‘was’ replaced with ‘were’ as requested.

Line 82: A space has been added in between 400m, now reads ‘400 m’.

Line 97: Changed to ‘eDNA-based’ as requested.

Line 116: Changed to ’50 mL’ as requested.

Line 118: Changed to '50 mL’ as requested.

Line 130: Changed to '0.22 µm’ as requested.

Line 130: Changed to '0.22 µm' as requested.

Line 131: Changed to '0.45 µm' as requested.

Line 132: Changed to '3.1 µm' as requested.

Line 140: Changed to '760 µL’ as requested.

Line 142: Changed to '580 µL' as requested.

Line 145: Changed to '37 °C’ as requested.

Line 146: Changed to '200 µL’ as requested.

Line 149: Changed to '20 °C’ as requested.

Line 151: Changed to 'DNA’ as requested.

Line 158: Changed to '2 mm’ as requested.

Line 161: Changed to 'was taken’ as requested.

Lines 164 and 165: Changed to '10%’ as requested.

Line 165: Changed to ‘121 °C’ as requested.

Line 169: Changed to '200 µL ATL and 20 µL PK’ as requested.

Line 170: Changed to '56 °C’ as requested.

Line 171: Changed to '200 µL’ as requested.

Line 171-172: ‘(Supplemental Information 1: Table S5)’ removed and added into results section at line 250 as follows: ‘Four primer/probe combinations were found to be species-specific for C. chinesis in that no cross-amplification was detected when tested with DNA isolated from various snail specimens collected at the Pevensey Levels and Stodmarsh NNR. Primer/probe combination 8 was found to be the most promising, was optimised and gave a limit of detection (LOD) and limit of quantification (LOQ) of 1x10-4 ng/µL (Supplemental file 2).’

The reviewer queried the presence of a Table S4, it is within the Suplemental Information 1 document and a page break has been added before each new table to make this clearer. Reference to Table S4 is made on line 124.

Line 184-185: ‘(Supplemental Information 1: Table S6’ removed and added into results section at line 250 as follows: ‘Snail specimens were identified by Sanger sequencing followed by BLAST analysis (Supplemental Information 1: Table S5) and a subset chosen for assay specificity testing.’

Line 189: References added for BOLD and Genbank as requested

- Sayers et al 2020
- Ratnasingham and Hebert 2007

Line 192: Reference added for PrimerBLAST: Ye et al 2012

Line 198-199: The concentrations of the primers were optimized by independently varying their concentrations and the best results were achieved when different concentrations of each were used.

Line 205: Changed to ’56.3 °C’ and ’95 °C’ as requested.

Line 206: Changed to '95 °C’ and ’56.4 °C’ as requested.

Line 211: Changed to '1 ng/µL to 1x10^-6^ ng/µL’ as requested.

Line 221: Changed to ‘3 µL’ as requested.

Line 224: Changed to '0.08 ng/µL’ and ‘1 µL’ as requested.

Line 225: as for Line 198-199, when the assay was developed the primer concentrations were optimized and these gave the best results. Primer concentrations should optimised for every new PCR assay carried out.

Line 229: Changed to ‘0.08 ng/µL’ as requested.

Line 230: Changed to 'ddH_2_O’ as requested.

Line 263 and Line 272: Changed to '*C. chinensis*’ as requested.

Line 289: Some text has already been added at line 250 to state the results of the *in silico* and *in vitro* evaluation of assays which was previously missing as follows: ‘Snail specimens were identified by Sanger sequencing followed by BLAST analysis (Supplemental Information 1: Table S5) and a subset chosen for assay specificity testing. Four primer/probe combinations were found to be species-specific for *C. chinesis* in that no cross-amplification was detected when tested with DNA isolated from various snail specimens collected at the Pevensey Levels and Stodmarsh NNR. Primer/probe combination 8 was found to be the most promising, was optimised and gave a limit of detection (LOD) and limit of quantification (LOQ) of 1x10-4 ng/µL (Supplemental file 2).’

Line 292: Changed to ‘204 bp’ as requested.

Line 298, 300 and 303: Changed to ‘ *in silico*’ and ‘*in vitro*’ as requested.

Line 310: Changed to ‘400 m’ as requested.

Line 319: Changed to '138 m’ as requested.

Line 321: Changed to 'five’ as requested.

Line 330: Changed to '2021, and 6/18…’ as requested.

Line 345: Changed to '0.45 µm’ and ‘3.1 µm’ as requested.

Line 346: Changed to ‘six’ as requested.

Line 347: Changed to ‘0.22 µm’ and ‘two’ as requested.

Line 364: Changed to '*C. chinensis*’ as requested.

Line 375: Deleted ‘degradation’ and left ‘degraded’.

Lines 559-570: These references have been removed from the references and referred to as Willing 2021a [unpublished]; Willing 2021b [unpublished]; and Willing 2022 [unpublished] to differentiate between the three different reports. Additionally, a reference, Measures et al 2022 has also been removed from the references and referred to as Measures [unpublished].

Line 601: Changed to ‘p(-), that is’ as requested.

Table 3: ‘04874’ changed to ‘0.74’ as requested.

Figure 2: Changed to ‘*C. chinensis*’, comma added and parentheses removed as requested.

Figure 3: Changed to ‘*C. chinensis*’, comma added and parentheses removed as requested.

**Reviewer 2: line numbers refer to original manuscript.**

Line 13: I think the year is off by 1 year. Gray 1834 instead of 1833 – at least that is what is in the citation.

*Line 13: this has been amended to 1834.*

Line 94-96: Check for typos and grammar. Also, common names are not capitalized (with location exceptions). I have noticed that there are typos in the manuscript with a few instances of the species not being italicized (be sure to check your figure legends).

*Line 94-96: Common names have been amended so that they are not capitalized, and punctuation added.*

*The rest of the manuscript has been thoroughly checked and also edited as per reviewer 1s comments which covers the instances of species not being italicized mentioned.*

Line 121: For every site visited?

*Line 121: only one field blank was taken, the whole area is not very large and the sampling sites were not that far apart therefore we felt that it was reasonable to only take one field blank. I have tried to make clearer by adding ‘was also filtered at the Pevensey Levels ditch system’.*

What is ADAS and how many staff were there doing this work?

*ADAS is an environmental consultancy where Helen Rees and Ben Maddison are currently employed. 1 ADAS staff member and 2 Natural England staff carried out the sampling in 2021; and 2 ADAS staff members and 6 Natural England staff carried out the sampling in 2022. I’m not sure that adding the number of samplers adds anything to the manuscript so I have not added this into the text.*

*We noticed that we have used both ADAS and RSK ADAS and we have amended to the latter in case this added to the confusion as to what ADAS is.*

Typically, the catalog number of associated filters is stated so someone else could buy the exact filters to repeat the assay.

*The product number of the filters has been added at line 119.*

Line 140: What is ATL buffer? What concentration of proteinase K?

*The ATL buffer is within the DNeasy blood and tissue kit (Qiagen) and its makeup is not stated so I am unable to add this information but it is a lysis buffer. The concentration of PK is not stated by the manufacturers so cannot be added, both are obviously proprietary information. I have added that both ATL and PK are part of the DNeasy blood and tissue kit (Qiagen).*

Line 196: Including a supplementary table of the species chosen would be helpful as other researchers may want to see what species those primers would likely not amplify. Were any of these the sister species? How did you determine what was a closely related species? Please include a citation?

*The species chosen for cross-reactivity testing are shown within Table S5 and are marked with an ‘*’, I have added a reference to this at line 196. Only UK species were used for cross-reactivity testing. I have removed the ‘closely-related’ from the text.*

Line 263: Species needs to be in italics

*Line 263: This has been done.*

Line 304: Does this mean that you all sequenced the positive samples that came from the qPCR assay and the results were the correct species? If so, I think that shows that is assay is working well and it needs to go in your methods and results section. If not, then I would recommend going back to those positive eDNA samples and sequencing them to be sure you are not amplifying another organism. Quite often in real-world sampling (field samples) I have noticed eDNA assays go out the “window” because they were amplifying the wrong target. Sometimes it is not even closely related to the target organism.

*A selection of positive amplifications were sequenced as to sequence all of the positive amplifications would not have been feasible within the budget for the project. I have amended the text to reflect this at line 304. Some information on this has been added to the methods and results sections. At line 219 (methods) the following text was added ‘A selection of positive amplification were Sanger sequenced and returned sequences and identified using BLAST.’. At line 277 (results) the following text was added ‘In order to confirm the presence of C. chinensis a selection of positive amplifications were Sanger sequenced and analysed using BLAST. All the positive amplifications sequenced were determined to be C. chinensis laeta as were most of the collected C. chinensis specimens with the remainder being identified as C. chinensis (Supplemental Information Table S5).’*

Additional Notes:

For the map figures I suggest making a map that shows 1) where eDNA samples were taken and if they were positive 2) where snails were collected and if they were positive for C. chinensis. Maybe a pie chart graphic or something to make it clear where the snails were found and if the eDNA assay was positive from that site. From my point of view it is less concerning to find positive eDNA, but no snails compared to negative eDNA and finding snails. It would suggest that the assay is not sensitive enough. There are times where you might not be able to find the organism, but rather “traces” (eDNA) of the organism. The authors made the point that there is room for improvement, but I think what you have shown suggests there is room for improvement when it comes to sensitivity assuming the authors sequenced the positive qPCR samples.

*Figures 2 and 3 already show the locations of where the eDNA samples were taken but we have added snail icons to the figure to show where C. chinensis specimens were collected/seen whilst traditional surveying was performed (specimens were not collected from every location that they were seen). The reviewer is correct that the assay is probably not sensitive enough and further work will be required to improve this as we did sequence a selection of the positive PCR amplicons.*

When writing a paper about the development of an assay every detail (even if it seems extra) matters to someone who would want to repeat the assay in future. I also suggest adding more details in the materials and methods to make it clearer people who may use this assay in the future.

*When writing this manuscript, Thalinger et al 2021 (Helen Rees was a co-author of this publication) was used as a guide for how much information to include in the methods section. We feel that sufficient information has been included and are not sure what else can be added. When performing the analysis for the Thalinger paper we looked at a checklist containing 122 variables to determine the validation level of an assay. We used this list of variables as a guide for assay development and having gone back through this list we have reported everything that we are able to e.g. we cannot report variables for ethanol precipitation of samples and we also have not looked at eDNA shedding rates or transport which would take the assay to a level five validation as that was not within the scope of the project. We do feel that the paper meets the criteria for level 3 to level 4 in terms of validation.*

The locations/names of the sites the authors visited might be clear to the authors, but not clear to someone else who may also want to visit these sites. Please include GPS coordinates in your tables.

*Tables S1-S4 in Supplemental Information 1 already contain either GPS or What3words locations. Text has been added to 110 to make it clear that sample information can be found within Supplemental File 1 Table S1. ‘All sample information can be found in Supplemental File 1, Table S1.’ Mention of Tables S2 and S3 is already found at lines 114-115, however this sentence has been edited to the following: All sample information can be found in Supplemental File 1 Table S2 and Table S3). Mention of Table S4 is already found at line 124.*
